# Efficacy of Myopia Control and Distribution of Corneal Epithelial Thickness in Children Treated with Orthokeratology Assessed Using Optical Coherence Tomography

**DOI:** 10.3390/jpm12020278

**Published:** 2022-02-14

**Authors:** Yu-Kai Kuo, Yen-Ting Chen, Ho-Min Chen, Pei-Chang Wu, Chi-Chin Sun, Ling Yeung, Ken-Kuo Lin, Hung-Chi Chen, Lan-Hsin Chuang, Chi-Chun Lai, Yau-Hung Chen, Chun-Fu Liu

**Affiliations:** 1 Department of Ophthalmology, Chang Gung Memorial Hospital, Keelung 20401, Taiwan; ken0975515036@gmail.com (Y.-K.K.); cornerbugi@gmail.com (H.-M.C.); arvinsun@cgmh.org.tw (C.-C.S.); lingyeung@gmail.com (L.Y.); lanhsin.chuang@gmail.com (L.-H.C.); chichun.lai@gmail.com (C.-C.L.); 2 College of Medicine, Chang Gung University, Taoyuan 33302, Taiwan; gradychen1107@gmail.com (Y.-T.C.); wpc@adm.cgmh.org.tw (P.-C.W.); d12093@cgmh.org.tw (K.-K.L.); mr3756@cgmh.org.tw (H.-C.C.); 3 Department of Ophthalmology, Chang Gung Memorial Hospital, Linkou 33305, Taiwan; 4 Department of Ophthalmology, Kaohsiung Chang Gung Memorial Hospital, Kaohsiung 83301, Taiwan; 5 Center for Tissue Engineering, Chang Gung Memorial Hospital, Linkou 33305, Taiwan; 6 Department of Chemistry, Tamkang University, Tamsui 251301, Taiwan; yauhung@mail.tku.edu.tw; 7 Program in Molecular Medicine, National Yang Ming Chiao Tung University, Taipei 112304, Taiwan

**Keywords:** axial length changes, anterior segment OCT, corneal epithelial thickness, myopia control, orthokeratology

## Abstract

The association between myopia control efficacy in children treated with orthokeratology and corneal epithelial thickness is still unknown. The aim of this study was to explore the corneal epithelial thickness and its association with axial length changes in children treated with orthokeratology. This retrospective cohort study enrolled children aged from 9 to 15 years who had received orthokeratology for myopia control and had been followed up for at least 1 year. Anterior segment optical coherence tomography was performed to generate wide epithelial thickness maps of the patients. Annual axial length changes were calculated from the axial length at 6 months after the initiation of orthokeratology lens wear and at final measurements. Corneal epithelial thickness data were obtained from 24 sectors and a central 2 mm zone of the wide epithelial thickness map. Associations between annual axial length changes and corneal epithelial thickness for each sector/zone of the wide epithelial thickness map, and orthokeratology treatment data were determined by generalized estimating equations. Finally, a total of 83 eyes of 43 patients (mean age 11.2 years) were included in the analysis. The mean annual axial length change was 0.169 mm; when regressing demographic and ortho-k parameters to mean annual axial length changes, age and target power were both negatively associated with them (β = −14.43, *p* = 0.008; β = −0.26, *p* = 0.008, respectively). After adjusting for age and target power, the annual axial length changes were positively associated with the corneal epithelium thickness of IT1, I1, SN2, and S2 sectors of the wide epithelial thickness map, and negatively with that of the I3 sector. In conclusion, we identified associations between annual axial length changes and the corneal epithelium thickness of certain sectors in children treated with orthokeratology. This may facilitate the design of orthokeratology lenses with enhanced efficacy for myopia control.

## 1. Introduction

In recent years, orthokeratology (ortho-k) of different geometries has made it possible to correct not only myopia but also hyperopia, astigmatism, and presbyopes [1]. In common clinical practice, ortho-k is an alternative to eyeglasses and soft contact lenses for optical correction in patients with mild to moderate myopia. If the reverse-geometry, rigid gas-permeable lenses are worn overnight, ortho-k can temporarily flatten the central cornea and steepen the peripheral cornea, allowing myopia lens wearers to have sufficient visual acuity during the daytime [1,2]. These lenses have been demonstrated to be effective and safe for overnight wear with rigorous sanitary compliance [3]. Surprisingly, recent studies have also reported retardation of myopia progression [2]. On average, ortho-k decreases the elongation of axial length (AL) by approximate 50%, having a comparable effect on myopia control to low-dose atropine [1,2,4].

The major change after ortho-k lens wear is the remodeling of the corneal epithelium, presenting as a typical bull’s-eye pattern with stability attained as a result of regular usage for 1 to 3 months [5,6,7,8]. The changes in the corneal epithelial thickness (CET) include central epithelium thinning and midperipheral epithelium thickening, which may contribute to peripheral myopic defocus of the retina. Although the mechanism of action of ortho-k on myopia control is still debatable, animal studies have found that relative peripheral myopic defocus may retard axial elongation [9,10,11]. Hence, the induction of peripheral myopization may be the effect of ortho-k in myopia control [12].

With the development of anterior segment optical coherence tomography (AS-OCT), a noncontact technique for high-resolution cross-sectional imaging, we can accurately measure the epithelial changes in corneal tissues [13,14]. The newly available wide-field pachymetry scan pattern of the spectral-domain optical coherence tomography (SD-OCT) device (RTVue-XR Avanti, Optovue Inc., Fremont, CA, USA) has the ability to determine three-dimensional corneal epithelial structures of up to 9 mm-diameter and generate a wide epithelial thickness map (ETM) [13].

It is accepted that the mechanism by which ortho-k reduces myopia progression is relative peripheral myopization. This effect may be attributed to CET changes, which can be better evaluated using the 9 mm-diameter wide ETM [4,15]. Therefore, we hypothesized that the CET of wide ETM sectors, especially those from the midperiphery, are associated with myopia control efficacy in children treated with ortho-k. To test this hypothesis, we investigated the CET of each sector/zone of the wide ETM and their associations with AL changes.

## 2. Materials and Methods

In this retrospective cohort study, patients were enrolled from the ortho-k continuous follow-up (OKCFU) open cohort database, which includes patients who have received ortho-k at Chang Gung Memorial Hospital, Keelung, Taiwan since January 2015. The database contains complete medical records, including a series of detailed examinations before and after the initiation of ortho-k treatment.

The inclusion criteria were as follows: (1) patients aged from 9 to 15 years; (2) ortho-k treatment administered for myopia control; (3) at least 12 months of follow-up after initiating ortho-k; and (4) ortho-k brand DreimLens^®^ (Brighten Optix, Taipei, Taiwan) used.

The exclusion criteria were as follows: (1) presence of any other ocular diseases besides myopia; (2) history of ortho-k use for less than 90% of all nights during the follow-up period; (3) development of any ortho-k-related complications; (4) discontinuation of ortho-k for more than 2 weeks during the follow-up period; (5) previous prescription of other ortho-k lenses; (6) combination with other myopia control methods; and (7) insufficient data for the wide ETM for at least 6 months or AL measurement for at least 12 months after the initiation of ortho-k treatment.

The studied patients were all treated with DreimLens^®^ (Brighten Optix), including anastigmatic-design and astigmatic-design lenses. The ortho-k lenses used in the current study were reverse-geometry design, rigid gas-permeable lenses (Boston XO material by Brighten Optix Co., Taipei, Taiwan). The oxygen permeability is 140 DK Units (gas to gas method). The overall diameters of the prescribed lenses were 10.2–10.8 mm. The back optic zone diameter was 6 mm; the reverse curve radius was 0.6 mm; the alignment curve radius was 1.1–1.4 mm, and the peripheral zone width was 0.4 mm. The prescription included only target power, diameter, and alignment curve (two alignment-curve powers for an astigmatic-design lens). The target power was prescribed using the full correction power measured by an auto refractometer (ARK-1a; Nidek, Aichi, Japan) with trial ortho-k lens wearing under cycloplegic conditions. The target power was the sum of measurement power (vertex distance corrected) and trial lens power. Other lens design parameters were not adjusted.

All patients received scheduled ocular examinations before and after wearing ortho-k lenses. All ocular examinations were performed by experienced ophthalmologic technicians under C-F Liu’s supervision. Pre-treatment tests included best-corrected visual acuity (BCVA), cycloplegic refractive power/corneal power measurement (Auto Ref/Keratometer ARK-1a; Nidek), and AL measurement (IOL Master^®^ 500; Carl Zeiss Meditec AG, Jena, Germany). Post-treatment tests included uncorrected and best-corrected visual acuity and slit-lamp biomicroscopy before and after fluorescence staining of the cornea during each follow-up. AL was measured every 6 months, and AL at 6 months after the initiation of ortho-k lens wear was used to calculate the annual AL change because of fluctuations of AL at the beginning of ortho-k treatment [16]. AS-OCT (RTVue-XR Avanti, Optovue Inc.) with a 9 mm wide-field pachymetry scan was performed at least 6 months after the initiation of ortho-k lens wearing and as needed, based on the physicians’ experience. The latest measured wide ETM obtained from AS-OCT of each patient was collected for analysis.

### 2.1. Corneal Epithelial Thickness Map

Maps were generated using a spectral-domain AS-OCT (RTVue-XR Avanti) with the corneal adaptor module and the wide-field pachymetry scan pattern (PachymetryWide scan, 8 radial lines with 9 mm scan length, 1056 axial scans each radial line, repeated 4 times, RTVue-XR software version 2017.1.0.151). The wavelength was set at 830 nm, providing an axial resolution of 5 μm. The CET was calculated as the distance between the air–tear interface (first curve) and the epithelium–Bowman’s layer boundary (second curve). If the first and second curves did not fit the real corneal contour, OCT images were rechecked and a manual adjustment was made according to the manufacturer’s guidelines.

**Figure 1:**
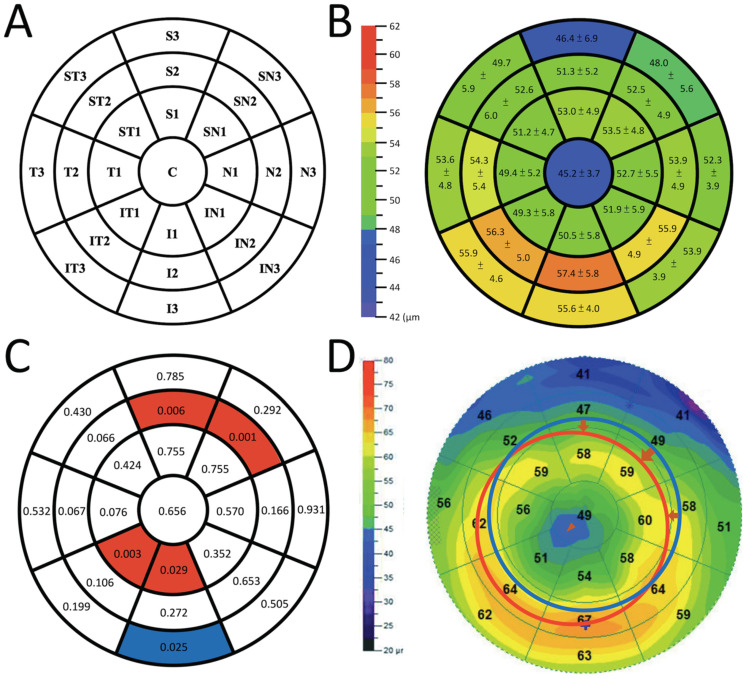
The illustrations regarding the wide epithelial thickness map (ETM) in the current study. (**A**) A 9 mm-diameter wide ETM and the nomenclature of each sector/zone (right eye). (**B**) The mean epithelial thickness (±standard deviation) of each sector/zone on the wide ETM. (**C**) Associations between annual changes in axial length and corneal epithelial thicknesses of the wide ETM sectors, presented with *p*-values calculated using generalized estimating equations. Associations in sectors IT1, I1, SN2, and S2 were significant with positive β coefficients (red sectors), while sector I3 was significant with a negative β coefficient (blue sectors). (**D**) Demonstration of the pupil centered wide ETM from a patient with inferotemporal shift and tilting of orthokeratology. The midperipheral steepening moves inferotemporally (red circle) from its centered location (blue circle), and the central treatment zone also moves inferotemporally (arrowhead).

A 9 mm-diameter wide ETM was generated and divided automatically by the system into a central 2 mm-diameter circle and three surrounding rings, which were denoted as C zone (central 2 mm circular zone), Ring 1 (paracentral 2–5 mm), Ring 2 (midperipheral 5–7 mm), and Ring 3 (peripheral 7–9 mm). Ring 1, Ring 2, and Ring 3 were further divided into eight sectors, namely superior sectors S1, S2, and S3; superior nasal sectors SN1, SN2, and SN3; nasal sectors N1, N2, and N3; inferior nasal sectors IN1, IN2, and IN3; inferior sectors I1, I2, and I3; inferior temporal sectors IT1, IT2, and IT3; temporal sectors T1, T2, and T3; and superior temporal sectors ST1, ST2, and ST3, as demonstrated in Figure 1A. The values of these 24 sectors and the C zone were recorded for further statistical analysis.

### 2.2. Statistical Analysis

All statistical analyses were conducted using SPSS version 26.0 for Mac (IBM, Armonk, New York, NY, USA). For the analysis of two eyes in one patient, the generalized estimating equations (GEE) model was applied to account for the dependency of the outcome for the two eyes. Independent working correlation and robust standard error were adopted to estimate the significance of parameters with the lowest Corrected Quasi-likelihood under the Independence Model Criterion (QICC). Age, gender, target power, diameter, alignment-curve power of ortho-k, astigmatism prescription, mean AS-OCT time, follow-up duration, AL at baseline, AL at 6 months after lens wear, and AL at final visit were analyzed to determine significance with annual AL changes. Annual AL changes were calculated as follows: (AL at final visit–AL at 6 months after lens wear)/days between the 6-month measurement and final visit × 365.25. Among these parameters, patient age and the target power of ortho-k were significant, and both were used as covariates when analyzing the associations between the CET of each sector/zone of the wide ETM and annual AL changes. A two-tailed *p*-value < 0.05 denotes a significant difference and no adjustment of α error was made.

## 3. Results

The medical records of children matching the inclusion criteria were extracted from the OKCFU database. Of the 56 children (109 eyes) enrolled, one was excluded because of overnight ortho-k use for less than 90% of all nights during the follow-up period, one was excluded because of suspected infection, one was excluded because of combination treatment with atropine, and 10 were excluded because of insufficient data of the wide ETM or AL measurement. Finally, 83 eyes of 43 children including 22 boys and 21 girls were analyzed. These studied patients all had uncorrected visual acuity exceeding 18/20 one month after the initiation of lens wear. Their mean age was 11.2 ± 1.9 (range, 9–15) years. Their mean AL at baseline was 25.2 ± 0.9 mm. The mean follow-up duration was 28.3 ± 12.1 months. The mean annual AL change was 0.169 ± 0.158 mm. The mean AS-OCT examination time point was 21.2 ± 11.8 months (earliest = 10 months) after the initiation of ortho-k lens wear. Other demographics and ortho-k parameters are summarized in Table 1.

The mean CET of each sector/zone is shown in Figure 1B, and their associations with annual AL changes (μm) are summarized in Figure 1C and Table 2. Sectors IT1, I1, SN2, and S2 were significant with positive β coefficients, while sector I3 was significant with a negative β coefficient.

## 4. Discussion

Ortho-k has become popular in recent years owing to its benefits in both myopic refractive error correction and myopia control for children. This study analyzed the associations between annual AL changes and the CET of wide ETM sectors/zones generated by AS-OCT, and found that sectors IT1, I1, SN2, and S2 were positively associated with annual AL changes, while sector I3 was negatively associated with annual AL changes (Figure 1C), after adjustment for age and target power. In current clinical practice, the age for the initiation of ortho-k treatment is a factor that cannot be manipulated, and target power can only be adjusted slightly to correct refractive error during the daytime. Hence, the determination of other factors that influence myopia control efficacy is of clinical importance. To the best of our knowledge, this is the first study using the wide-field pachymetry scan pattern of the AS-OCT to explore the associations between the CET of wide ETM sectors/zones and AL changes in school-aged children, using ortho-k lenses for myopia control.

It has been shown that regular overnight wearing of ortho-k lenses elicits central corneal flattening and midperipheral steepening [1,13,14]. Kim et al. demonstrated a consistent result obtained with the 6 mm-diameter ETM generated by the same model of spectral-domain OCT, showing that the mean changes in the CET of the central (2 mm in diameter), paracentral (2–5 mm annular ring), and midperipheral (5–6 mm annular ring) areas were −8.4, −1.4, and +2.7 μm, respectively [5]. The significant thickening of the epithelium in the midperipheral zones (*p* < 0.001) was thought to coincide with the reverse curve of the ortho-k lens [5].

Mild to moderate lens decentration is another common and inevitable phenomenon during ortho-k treatment [17]. Kim et al. reported that the decentration of the treatment zone was 0.25 ± 0.31 mm toward the temporal side, and 0.23 ± 0.40 mm toward the inferior side [5]. Chen et al. reported that 67% of their patients had inferotemporal decentration [17]. When inferotemporal decentration of ortho-k occurred, midperipheral steepening was also observed to shift inferotemporally (Figure 1D, i.e., midperipheral steepening was leaving sectors SN2 and S2, leading to the relative thinning of these sectors). The pressure from the lid and the compression from the shifted alignment curve can cause further thinning. In addition, the central treatment zone moves from C toward IT1 and I1, causing the thinning of IT1 and I1. Interestingly, the distribution of these thinned sectors (IT1, I1, SN2, S2) matches the distribution of the wide ETM sectors, with positive associations with annual AL changes in our analysis (Figure 1C,D). This suggests that the thinning of these wide ETM sectors, which leads to a slower axial length elongation, may be related to the inferotemporal shift of ortho-k.

The influence of ortho-k lens decentration on control of myopia remains controversial [18,19]. Wang et al. concluded that the decentering of the ortho-k lens < 1.5 mm could improve myopia control efficacy compared with a centric lens [18]. Moreover, Wu et al. [19] divided lens decentration into three groups: mild (<0.5 mm), medium (0.5–1.0 mm), and severe (>1.0 mm). The AL growth over 2 years in the severe decentration group was 0.23 ± 0.29 mm, which demonstrated better efficacy of myopia control compared with the medium and mild lens decentration groups. Our results may provide an explanation of the relationship between better myopia control and inferotemporal shift of ortho-k, that is consistent with the findings of the above studies [18,19]. However, further studies addressing ortho-k lens decentration based on visual axis, pupil center or corneal center, the magnitude of these decentrations, and their relationship with AL changes are needed to confirm this observation.

Higher-order aberrations (HOAs) may influence visual quality [20]. It has also been reported that ortho-k lens decentration was positively correlated with an increase in coma-like HOAs (*p* < 0.01), an increase in spherical-like aberration (*p* < 0.01), and a reduction in contrast sensitivity function (*p* < 0.0001) [21]. A significant increase in HOAs root mean square (RMS) (0.1 μm, *p* < 0.001) was also found after months of ortho-k lens wear [22]. These were considered as disadvantages yet to be alleviated. However, recent studies observed a considerable effect of increased HOAs on retardation of AL elongation [1,23,24]. Lau et al. reported a 0.1 mm/year decrease in AL elongation per 0.1 μm HOAs RMS [24]. Hiraoka et al. also observed a significant correlation of AL elongation with changes in coma-like aberration, spherical-like aberration, total HOAs, and corneal multifocality; among these, coma-like aberration was revealed to be the most relevant variable by multiple linear regression analysis [23].

Our study may link these findings, supporting the potential role of asymmetric HOAs in myopia control. When wearing an ortho-k lens, upper eyelid pressure may lead to lens decentration and tilting. It may also contribute to the change in epithelial thickness by decentration and the seesaw effect of ortho-k lens tilting [13]. Greater decentration from the pupil and tilting of the ortho-k lens may contribute to the thinner epithelium at IT1, I1, SN2, and S2, and the thicker epithelium at I3. They may cause more asymmetric HOA-like coma aberrations and total HOAs [21,22], which contribute to increases in corneal pseudo-accommodation, and ultimately lead to retardation of AL elongation [23,24]. These were detected from the distribution of the wide ETM sectors in the current study (Figure 1C,D), and gave a possible explanation of how the thinning of these sectors results in better myopia control. Nevertheless, lens decentration, tilting, and their influences on AL merit more future studies to identify.

Consistent with previous findings [1,25,26], the results of the current study revealed a negative association of age with annual AL changes. Furthermore, this study also found a negative association of target power with annual AL changes. This finding is also supported by the results of two prospective studies [23,25], and may be explained by greater midperipheral corneal changes under prescriptions of higher target power (greater peripheral myopization), resulting in slower AL elongation [4,15]. Therefore, age and target power were used as covariates in the statistical analysis to minimize their influence on AL changes.

Our study has some limitations. Data for AS-OCT before ortho-k treatment were inadequate because of the retrospective nature of the current study. It is possible that variations in baseline CET exist among individuals, while patients with better myopia control happened to have thinner corneal epitheliums in sectors IT1, I1, SN2, and S2. However, previously reported CET from children not wearing ortho-k lenses indicated that they had consistent CET in different sectors, ranging from 51.5 to 53.3 μm with small standard deviations [27]. After wearing ortho-k lenses, the CET was remodeled significantly in almost all sectors with a typical bull’s-eye pattern [5], and it was also the final remodeled CET that had the direct effect on peripheral myopic defocus and HOAs, influencing myopia control. Therefore, the shape of the cornea after ortho-k lens wear may be more important for evaluating myopia control efficacy.

Other limitations of this study include small sample size, retrospective design, and two-eyes enrollment, which was adjusted for by GEE analysis. The timing of AS-OCT measurement was not standardized but was performed at least 10 months after ortho-k lens wear. As the corneal surface has been reported to be stable after regular ortho-k usage for 1 to 3 months [6,7], and the lens decentration was also stable after 3 months [28], the measurement error from different timings may be minimal. Furthermore, pupil diameter may be an important factor for myopia control mechanisms [1]. However, pupil size changes with light intensity. Therefore, it should be measured by a standard method under strict environmental control. The current study is retrospective and cannot provide accurate pupil diameter data for further evaluation. Future prospective studies with standardized pupil measurement may resolve this limitation, and we are currently conducting a study addressing this issue.

## 5. Conclusions

After adjusting for age and target power, the annual axial length changes were positively associated with the corneal epithelium thickness of IT1, I1, SN2, and S2 sectors of the wide epithelial thickness map, and negatively with that of the I3 sector. The wide epithelial thickness map (ETM) generated by AS-OCT may provide useful information about myopia control efficacy. Epithelial structural information from the wide ETM may facilitate the design of ortho-k treatment with enhanced myopia control efficacy or assist personalized ortho-k treatment [29,30,31]. Further prospective researches addressing the limitations of the current study are required to confirm the current study results and may identify more factors contributing to the myopia control efficacy of ortho-k treatment.

## Figures and Tables

**Table 1:** Demographics and treatment data revealed that age and target power were both negatively associated with annual axial length changes.

	N = 83	β	*p*-Value ^a^
Age (years)	11.2 ± 1.9	−14.43	0.008 *
Gender (male:female)	22:21	−13.87	0.623
Target power (diopter)	3.28 ± 1.29	−0.26	0.008 *
Diameter (mm)	10.56 ± 0.13	<0.001	0.777
Alignment-curve power (diopter) ^b^	42.59 ± 1.21	0.04	0.691
Prescription astigmatism (eye)	Yes 27; No 56	−8.36	0.686
Mean AS-OCT exam time point (months) ^c^	21.20 ± 11.80	−1.36	0.186
Follow-up duration (months)	28.27 ± 12.09	−1.30	0.215
Axial length at baseline (mm)	25.08 ± 0.93	−23.81	0.137
Axial length at 6 months after lens wear (mm)	25.20 ± 0.91	−19.98	0.219
Axial length at final visit (mm)	25.46 ± 0.92	3.64	0.809
Annual axial length changes (μm/year)	168.86 ± 157.54		

Dependent variable: annual axial length changes (μm). AS-OCT: anterior segment optical coherent tomography; β: beta coefficient; N: number of eyes. ^a^ Correlation with annual axial length changes (μm/year) obtained using GEE. ^b^ Astigmatic-design lens power calculated using average alignment-curve powers. ^c^ Time duration between the initiation of orthokeratology and the latest AS-OCT measurement. * *p* < 0.05, using generalized estimating equation.

**Table 2:** Analysis between epithelial thickness and annual axial length changes.

Variable	Mean Thickness (μm)	β	95% CI for β		*p*-Value
			Lower	Upper	
Central Zone (2 mm)	45.2 ± 3.7	−1.162	−6.274	3.949	0.656
Ring 1 (2–5 mm)					
N1	52.7 ± 5.5	1.349	−3.302	6.000	0.570
SN1	53.5 ± 4.8	0.815	−4.311	5.941	0.755
S1	53.0 ± 4.9	0.751	−3.973	5.475	0.755
ST1	51.2 ± 4.7	1.616	−2.350	5.582	0.424
T1	49.4 ± 5.2	3.387	−0.355	7.128	0.076
IT1	49.3 ± 5.8	4.638	1.583	7.693	0.003 *
I1	50.5 ± 5.8	4.445	0.459	8.432	0.029 *
IN1	51.9 ± 5.9	2.234	−2.473	6.941	0.352
Ring 2 (5–7 mm)					
N2	53.9 ± 4.9	3.438	−1.426	8.301	0.166
SN2	52.5 ± 4.9	5.723	2.312	9.134	0.001 *
S2	51.3 ± 5.2	5.325	1.531	9.119	0.006 *
ST2	52.6 ± 6.0	3.774	−0.252	7.799	0.066
T2	54.3 ± 5.4	3.746	−0.265	7.757	0.067
IT2	56.3 ± 5.0	3.778	−0.799	8.355	0.106
I2	57.4 ± 5.8	2.092	−1.639	5.822	0.272
IN2	55.9 ± 4.9	0.981	−3.294	5.257	0.653
Ring 3 (7–9 mm)					
N3	52.3 ± 3.9	−0.269	−6.347	5.810	0.931
SN3	48.0 ± 5.6	1.970	−1.693	5.634	0.292
S3	46.4 ± 6.9	0.537	−3.317	4.391	0.785
ST3	49.7 ± 5.9	1.768	−2.625	6.161	0.430
T3	53.6 ± 4.8	1.931	−4.130	7.992	0.532
IT3	55.9 ± 4.6	−3.128	−7.907	1.650	0.199
I3	55.6 ± 4.0	−5.372	−10.069	−0.674	0.025 *
IN3	53.9 ± 3.9	−1.817	−7.155	3.521	0.505

Dependent variable: annual axial length changes (μm). β: beta coefficient; CI: confidence interval; S: superior sector; SN: superior nasal sector; N: nasal sector; IN: inferior nasal sector; I: inferior sector; IT: inferior temporal sector; T: temporal sector; ST: superior temporal sector. * *p* < 0.05, using generalized estimating equations with adjustment of age and target power together.

## Data Availability

Datasets generated and analyzed during this study are not publicly available due to patient privacy protection. Such data are available from the principal investigator on reasonable request. For data access requests on this retrospective cohort, please contact Chun-Fu Liu, Department of Ophthalmology, Chang Gung Memorial Hospital, Keelung, Taiwan, e-mail address: legendlcf@gmail.com.
